# Partial SAA patients benefit from delayed response of IST

**DOI:** 10.3389/fimmu.2023.1067977

**Published:** 2023-02-10

**Authors:** Ting Wang, Chaomeng Wang, Chunyan Liu, Zonghong Shao, Rong Fu

**Affiliations:** Department of Hematology, Tianjin Medical University General Hospital, Tianjin, China

**Keywords:** severe aplastic anemia (SAA), immunosuppressive therapy (IST), delayed response, cyclosporin A (CsA), eltrombopag (EPAG)

## Abstract

**Introduction:**

Severe aplastic anemia(SAA)is a severe disease characterized by immune-mediated bone marrow failure and pancytopenia. Immunosuppressive therapy (ATG plus CsA, IST) is the standard treatment for patients who are not suitable for allogeneic hematopoietic stem cell transplantation (allo-HSCT). Some patients have a delayed response after 6 months of ATG, and unnecessary to be given secondary ATG or allo-HSCT. We attempted to distinguish patients who may get potential delayed response from those who were really not responsive to IST.

**Methods:**

We collected data from 45 SAA patients who were assessed no-response to IST at 6 months after rATG and failed to receive secondary ATG or allo-HSCT.

**Results:**

CsA plus eltrombopag (EPAG) group has an extra 75% response rate while CsA maintenance group has an extra 44% response rate at 12 months. ATG was applied within 30 days after diagnosis, ATG dosage was suffificient (ATG/lymphocyte ≥2), and absolute reticulocyte count (ARC) was ≥30×109 /L at 6 months, indicated patients could get delayed response and benefifit from CsA maintenance. Addition of EPAG could give an even better response. Otherwise, secondary ATG or allo-HSCT treatment were recommended to be given immediately.

**Clinical Trial Registration:**

https://www.chictr.org.cn/searchproj.aspx, identifier ChiCTR2300067615.

## 1 Introduction

Severe aplastic anemia (SAA) is cause by T-cell-mediated destruction of hematopoietic stem and progenitor cells, which presents as pancytopenia and hypocellular bone marrow (1). For patients who are older, frail, or lack of a matched donor, immunosuppressive therapy (IST) including antithymocyte globulin (ATG) and Cyclosporin A (CsA) is the first-line standard therapy (2, 3). According to the aplastic anemia guidelines, 3 months and 6 months after ATG were recommended to evaluate IST response (4, 5). Many patients with no response or relapse at 6 months will receive secondary ATG or allo-HSCT in most research centers (6). However, we found that some SAA patients had delayed response even though they didn’t receive secondary ATG or allo-HSCT. If these patients can be distinguished from the ones who really need to get the second-line therapy, the treatment risk and extra financial burden could be avoided. Therefore we retrospectively analyzed SAA patients who failed to respond to IST at 6 months and were unable to receive secondary ATG or allo-HSCT for multifarious reasons, and summarized their clinical characteristics.

## 2 Design and methods

### 2.1 Patients

We enrolled 45 SAA patients who were with no-response at 6 months after IST, including CsA(3-5mg/kg/day and achieve the plasma concentration 150-250ng/ml) and rabbit ATG(rATG, 2.5-3.5mg/kg/day for 5days, Genzyme Polyclonals S.A.S), but failed to receive secondary ATG or allo-HSCT between January 2009 and January 2021 in the Department of Hematology, General Hospital of Tianjin Medical University. 25 patients were given only CsA as the maintenance therapy, while other 20 patients received CsA plus eltrombopag (EPAG) as the second line treatment. The initial dose of EPAG was 50mg/day, and then was adjusted according to the patient’s efficacy and side effects. Patients were followed up by 12 months after rATG. MDS phenotype, PNH clones, chromosome, FISH, next generation sequence (NGS) (**Figure S1 of supplementary documents**) were detected for all SAA patients. There were two patients with PNH clones, the PNH clone was less than 5%. At 6 months after rATG, no patients developed clonal evolution according to repeat examination. During the follow-up period, no patients developed into MDS or leukemia.

### 2.2 Methods

**Figure 1:**
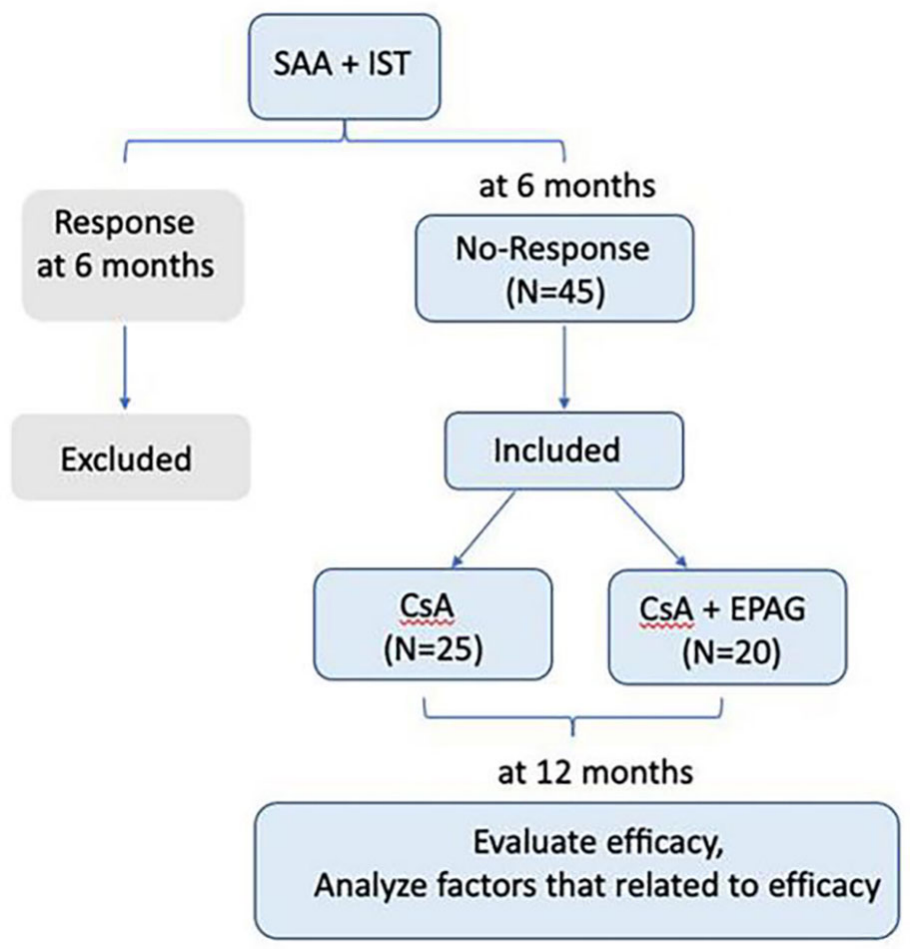
Process of the study.

All patients were diagnosed according to the guidelines of the International Aplastic Anemia Research Group (5). The criteria for SAA/VSAA were classified according to the guidelines of aplastic anemia (4, 5).Complete response (CR) was defined as hemoglobin concentration >100 g/L, neutrophil count >1.5×10^9^/L, and platelet count >100 × 10^9^/L, while partial remission (PR) was defined as transfusion independence and no longer meeting the criteria for SAA. In contrast, non‐responders (NR) still meet the criteria for SAA. The efficacy was evaluated at 12 months, the clinical characteristics and factors of efficacy in response group (CR+PR) and no-response group (NR) were analyzed retrospectively. The clinical data collection was approved by the Ethics Committee of Tianjin Medical University General Hospital (IRB2022-WZ-169). Our study process was shown in **Figure 1**.

### 2.3 Statistical analysis

Baseline characteristics were assessed by descriptive statistical analysis, including the mean and standard deviation (SD) or median (range) were presented as continuous variables and compared using the t test and Wilcoxon Mann−Whitney test. The frequency distribution (n, %) was presented as categorical variables and compared using the chi-squared test. Multivariate retrospective analysis was performed using logistic regression. All tests were two-sided, and a level of *P* < 0.05 served as the threshold for statistical significance in univariate analysis. *P* < 0.1 was considered the entry criteria for the model and used to identify an independent predictor for 12-month response to IST. All statistical analyses were performed in the SPSS statistical software package (SPSS version 21.0, IBM Corporation, Armonk, NY, USA).

## 3 Results

### 3.1 Patients’ characteristics

Among 45 AA patients with NR at 6months after rATG, 28 were (62.22%) SAA, 17 (37.78%) were VSAA at diagnosis. The median age was 24 (7–65) years old and 75.56% of patients were male. After 6 months, 20 patients (44.44%) were treated with CsA plus EPAG and 25(55.56%) were treated with CsA maintenance. The patients’ characteristics were shown in **Table 1**.

**Table 1:** Baseline clinical and laboratory characteristics.

Covariates	Total cohort (N=45)	RR (N=26)	NR (N=19)
Mean±SD/N50(N25, N75)/N (%)			
**Median age at diagnosis(range), years**	24(7-65)	25.5(8-65)	21(7-47)
**Median age at diagnosis (mean ± SD), years**	27 ± 13	29 ± 15	24 ± 11
**Gender,male/female**	34/11	20/6	13/6
Severity of AA,n. of patients(%)			
** VSAA**	17(37.78%)	7(26.90%)	10(52.60%)
** SAA**	28(62.22%)	19(73.10%)	9(47.40%)
ECOG			
** 0~1**	33(73.30%)	16 (61.50%)	17(89.50%)
** ≥2**	12(26.70%)	10 (38.50%)	2 (10.50%)
**WBC before IST,×10^9^/L**	1.56 ± 1.10	1.41 ± 0.89	1.66 ± 1.48
**ANC before IST,×10^9^/L**	0.20(0,0.44)	0.13(0,0.46)	0.30(0.03,0.43)
**ALC before IST,×10^9^/L**	74.42 ± 18.57	0.44 ± 0.69	0.61 ± 0.85
**ARC before IST,×10^9^/L**	9.51 ± 7.74	8.72 ± 5.35	8.40 ± 8.46
**RBC before IST,×10^12^/L**	2.28(1.91,2.87)	2.19(1.99,2.59)	2.35(1.68,3.14)
**HB before IST, g/L**	74.42 ± 18.57	74.65 ± 17.20	74.11 ± 20.79
**PLT before IST,×10^9^/L**	18.58 ± 14.05	19.16 ± 14.98	14.00 ± 6.59
**Myeloid in bone marrow (%)**	9.0(5.00,23.50)	10.25(5.75,26.50)	7.0(3.00,17.5)
**Erythrocyte in bone marrow(%)**	2.0(0.25,14.75)	3.0(1.00,14.13)	1.0(0,15.50)
**Megakaryocytes in bone marrow**	0	0	0

SAA, severe aplastic anemia; VSAA, very severe aplastic anemia; ATG, Anti-human thymocyte immunoglobulin; ANC, absolute neutrophil count; ALC, absolute lymphocyte count; ARC, absolute reticulocyte count; WBC, write blood cell; RBC, red blood cell; PLT, platelet.

### 3.2 Hematologic responses of SAA patients from 6 to 12 months

**Figure 2:**
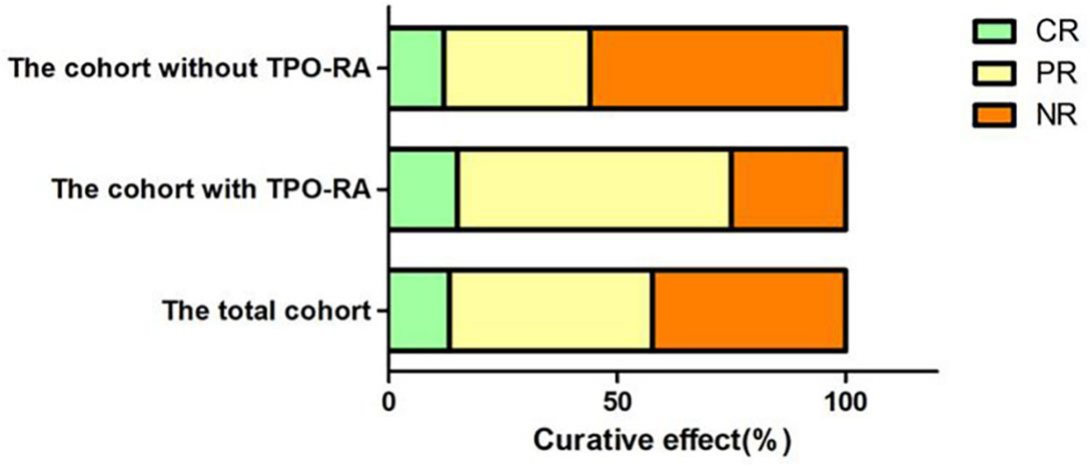
The response rate at different groups of aplastic anemia patients.

We followed up all these 45 SAA patients from 6 to 12 months. The extra response rate (CR+PR, RR) was 57.78% (26/45), no-response rate (NR) was 42.22% (19/45). In CsA plus EPAG group, 75% (15/20) got response at 12 months, and 25% (5/20) still with NR. In CsA group, 44% (11/25) got response at 12 months, 56% (14/25) didn’t get response, with statistical significance between the two groups (*P*=0.036) (**Figure 2**).

### 3.3 Factors that related to the efficacy from 6 to 12 months after rATG

#### 3.3.1 Factors that related to the efficacy from 6 to 12 months after rATG in CsA group.

The indicators were compared between RR and NR groups, including gender, age, ECOG score, severity of disease, rATG dose and the immune indicators before rATG and 6 months after rATG. Differences were found as showed in **Table 2**. No other meaningful comparisons were found (**Supplementary Table 1**).

**Table 2:** Factors related to the efficacy from 6 to 12 months after rATG with univariate analysis in CsA group.

Covariates	RR	NR	T/Z/χ2	*P* value
**N (%)**	11(44.00%)	14(56.00%)		
**rATG dosage/ lymphocytes** **(mg×kg^-1^/1×10^-9^)**			4.26	0.04^*^
** <2**	3(27.30%)	8(57.10%)		
** ≥2**	8(72.70%)	6(42.90%)		
**Days from diagnosis to IST**			9.29	0.002^*^
** <30days**	10(90.90%)	3(21.40%)		
** ≥30days**	1(9.10%)	11(78.60%)		
**CD8^+^T cells before IST, ×10^9^/L**	0.46 ± 0.37	0.80 ± 0.18	-2.93	0.008^*^
**mDC /pDC before IST**	0.67(0.50,1.44)	1.67(1.29,2.70)	-2.82	0.005^*^
**HB at 6 months after IST, g/L**	69.73 ± 7.59	57.33 ± 9.29	2.78	0.011^*^
**RBC at 6 months after IST,×10^12^/L**	2.62 ± 0.43	1.84 ± 0.37	2.001	0.057
**ARC at 6 months after IST,×10^9^/L**			7.35	0.007^*^
** <30**	1(9.10%)	10(71.40%)		
** ≥30**	10(90.90%)	4(28.60%)		
**megacaryocyte at 6 months after IST**	10.5(4.0,23.75)	3.0(0,12.00)	-2.82	0.005^*^

ATG, Anti-human thymocyte immunoglobulin; ALC, absolute lymphocyte count; mDC, myeloid dendritic cell; pDC, plasmacytoid dendritic cell; ARC, absolute reticulocyte count; HB, hemoglobin; red blood cell; PLT, platelet; * means P<0.05.

##### 3.3.1.1 The time from diagnosis of SAA to initiation of rATG treatment

The median time from diagnosis to rATG treatment was (19.10 ± 5.49) days in RR group and (50.08 ± 45.32) days in NR group (*P*=0.022). In addition, the extra response rate with the time less than 30 days (76.92%) was significantly higher than those over 30 days (8.33%) (*P* =0.002).

##### 3.3.1.2 rATG dosage

The dosage of rATG was (4.28 ± 0.78) mg/kg.d in RR group and (3.59 ± 0.99) mg/kg.d in NR group, with no statistical difference between them (*P*=0.11). Then we compared the relative dose of ATG and lymphocytes [ATG dose(mg/kg)/ALC (×10^9^/L)], the ratios were 2.80 (3.32, 8.18) vs 1.73 (1.64, 2.49) in RR and NR groups, respectively, with significant statistical difference (*P*=0.014). The response rate of patients with the ratio less than 2.0 (27.27%) was significantly lower than those with the ratio more than 2.0 (57.14%) (*P* =0.04).

##### 3.3.1.3 The CD8^+^T cells counts before IST

The CD8^+^T cells counts before IST was significantly lower in RR group (0.46 ± 0.37) ×10^9^/L than that in NR group (0.80 ± 0.18) ×10^9^/L, (*P*=0.008).

##### 3.3.1.4 The ratio of mDC/pDC before IST

The initial ratio of myeloid dendritic cell (mDC) and plasmacytoid dendritic cell (pDC) (mDC/pDC ratio) was 0.67(0.50,1.44)in RR group and 1.67(1.29,2.70)in NR group, with statistical difference(*P*=0.005).

##### 3.3.1.5 Indicators at 6 months after rATG

The indicators of bone marrow, peripheral blood and immune indexes at 6 months after rATG was compared between RR and NR groups. We found absolute reticulocyte count (ARC) at 6 months in RR group were significantly higher than it in NR group, which were (63.53 ± 26.19)×10^9^/L vs (27.30 ± 19.68)×10^9^/L respectively, (*P*=0.029). The hemoglobin at 6 months was higher in RR group (69.73 ± 7.59) g/L than it in NR group (57.33 ± 9.29) g/L, (*P*=0.011). The numbers of megakaryocyte in bone marrow at 6 months were significantly more in RR group 10.5 (4.0, 23.75) than it in NR group 3.0 (0, 12.0), (*P*=0.005).

#### 3.3.2 Factors that related to the efficacy from 6 to 12 months after rATG in CsA plus EPAG group

Twenty SAA patients with no-response to IST at 6 months received CsA plus EPAG treatment, the indicators were compared in these patients between RR and NR groups at 12 months after rATG. Univariate analysis showed that ALC at diagnosis and the ratios of CD4/CD8 at 6 months after ATG were significantly different between these two groups. No other significant comparable indicators were found. (**Supplementary Table 2**).

##### 3.3.2.1 Impact of ALC before IST

The values of ALC in the RR group were 1.61(1.52, 1.65) (×10^9^/L) at diagnosis which were significantly lower than that in the NR group 1.76(1.71, 1.87) (×10^9^/L) (*P*=0.001).

##### 3.3.2.2 The ratio of CD4/CD8 at 6 months after IST

The ratio of CD4/CD8 at 6 months after IST was (1.68 ± 1.18) in the RR group, which was significantly higher than that in the NR group (0.37 ± 0.35), (P=0.03).

#### 3.3.3 Factors that related to the efficacy from 6 to 12 months after rATG in all patients

Cause the number of SAA patients in neither CsA group nor CsA plus EPAG group were sufficient to make multivariate analysis.We compare the indicators that may influence the efficacy at 12 months in all patients. In univariate analysis, TPO-RA added (*P*=0.04), Days from diagnosis to ATG <30 (*P*=0.03), lower of CD8^+^T cells count before IST (*P*=0.03), higher level of HB at 6months (*P*=0.03), higher ARC at 6months (*P*=0.003) predict a better response rate at 12 months. However only Days from diagnosis to ATG (*P*=0.04) and ARC at 6 months (*P*=0.020) was found associated with the efficacy at 12 months in multivariate analysis. (**Table 3** and **Supplementary Table 3**).

**Table 3:** Factors related to the efficacy from 6 to 12 months after rATG with univariate and multivariate analysis in all patients.

Covariates	P value	
	Univariate	Multivariate
**Eltrombopag**	0.04^*^	0.44
** Yes**		
** No**		
**rATG dosage/lymphocytes count(mg×kg^-1^/×10^9^)**	0.079	0.31
** <2.0**		
** ≥2.0**		
**Days from diagnosis to IST**	0.03^*^	0.04*
** <30**		
** ≥30**		
**CD8^+^T cells before IST, ×10^9^/L**	0.03^*^	0.26
**HB at 6months after IST,g/L**	0.03^*^	0.72
**ARC at 6months after IST,×10^9^/L**	0.003^*^	0.02*
** <30**		
** ≥30**		

* means P<0.05.

### 3.4 Predictors of the effect

**Figure 3:**
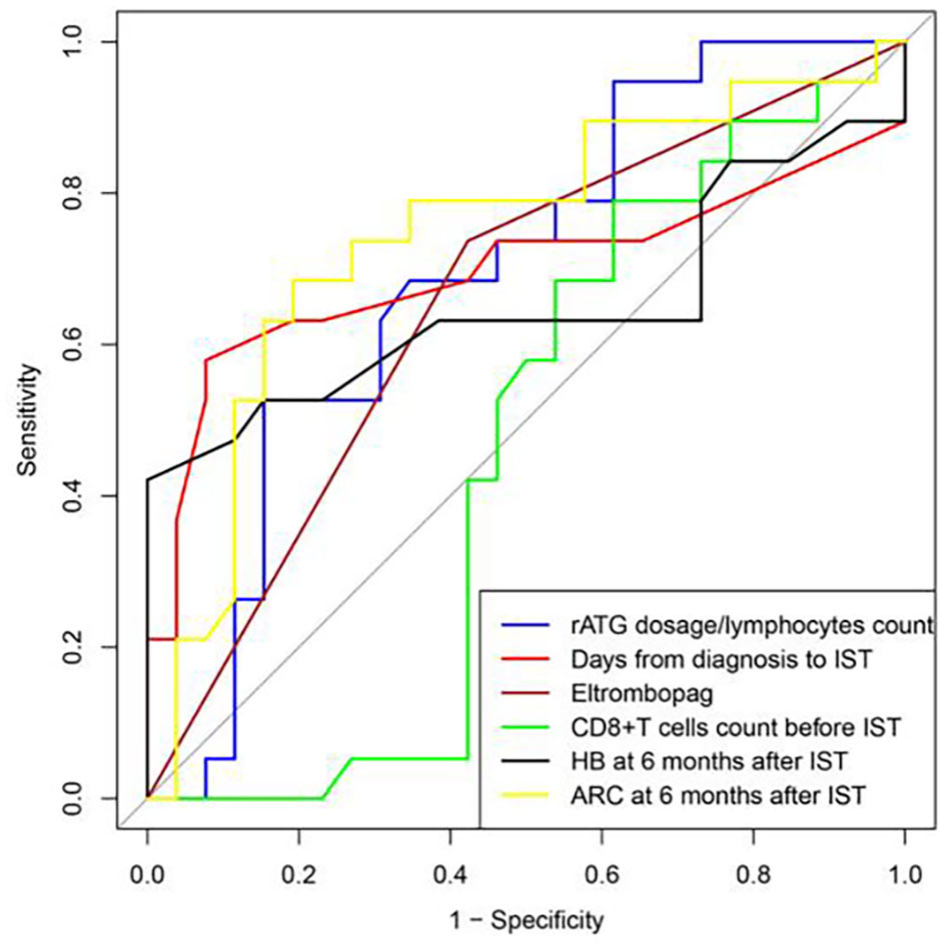
ROC curve was used to evaluate the predicting factors that influences the effect of IST at 12 months, ARC at 6 months after IST with AUC more than 0.7.

ROC curve was used to evaluate the predicting factors that influences the effect of IST at 12 months if the indexes had P-value lower than 0.1 in univariate studies or had a possible impact on the efficacy. ARC at 6 months after IST with AUC more than 0.7. The optimum critical value of ARC was 30.58×10^9^/L at the maximum of the Youden index. The sensitivity and specificity of ARC percentages were 68.40% and 80.80%. (**Figure 3**, **Table 4**).

**Table 4:** Prediction performance of the ROC analysis model.

index	AUC	Sensitivity (%)	Specificity (%)	The optimum critical value	Youden index
**rATG dosage/lymphocytes count(mg×kg^-1^/×10^9^)**	0.693	52.60	84.60	1.76	0.372
**Days from diagnosis to IST**	0.697	57.90	92.30	32.00	0.502
**Eltrombopag**	0.657	73.70	57.70	0.50	0.314
**Initial CD8^+^Tcells count**	0.460	78.90	38.50	0.90	0.174
**HB at 6 months after IST**	0.651	42.10	100.00	60.50	0.421
**ARC at 6 months after IST**	0.746	68.40	80.80	30.58	0.492

## 4 Discussion

For patients who are older, frail, or lack of a suitable donor, IST including ATG and CsA was recommended as the first line treatment for SAA patients. Normally SAA patients were evaluated every 3 months after ATG therapy according to the guidelines, the secondary ATG or allo-HSCT were recommended for patients who failed to response to IST at 6 months (5). In our study, we found even though the SAA patients with no-response at 6 months after rATG, if the patients kept receiving CsA treatment, 44% of them still got response at 12 months. If EPAG was added, an extra 75% patients got response at 12 months. The result demonstrated that some SAA patients have delayed response after IST. It is meaningful to distinguish this part of patients from real no-response patients, because this work can help avoiding overtreatment for delayed-response patients.

In our study, all SAA patients received rabbit ATG.We are not quite sure about if all kinds of ATG leading delayed response, maybe it is caused by ATG species. Many studies found that horse ATG (hATG) had a much rapid response rate than rabbit ATG, with a better RR at 3 and 6 months, but similar RR at 9 and 12 months (7, 8).This was consistent with our results that rATG may produce stronger and longer immune suppression with slower recovery of peripheral blood and bone marrow. Presumably, the above phenomena may be caused by different half-life time of ATG, for hATG 5.7 days and rATG 30 days. After the delayed response was confirmed, next we try to identify which part of patients were likely to experience a delayed response.

Multiple clinical studies have shown that the response rate to IST is associated with the interval time between diagnosis and ATG initiation (9, 10). ATG treatment within 30 days after SAA diagnosis was an independent prognostic factor, and the overall survival (OS) was significantly better than that of patients treated with ATG after 30 days (11). Our results also showed similar result that although the patients failed to response at 6 months after ATG, they still had a better RR at 12 months if they received rATG in 30 days after diagnosis when comparing with the ones who didn’t receive ATG in time. The result emphasized again early initiation of ATG therapy is extremely important for the novel SAA patients.

AA is an immune-mediated bone marrow failure disease. IST is at the core position. An adequate IST predicts a good response. In IST for SAA, ATG dosage was calculated only depends on patients’ weight, however in HSCT field, it is showed that the clearance rate of ATG was influenced by lymphocyte count of the recipient, the generation of anti-ATG antibodies and the time of transplantation and individual biodegradability. Does a relative insufficient ATG exist in SAA treatment? We further analyzed the relative ATG dose (ATG dosage/ALC (×10^9^/L)). Our results showed that the higher ATG dose which meant relative adequate immunosuppressive therapy produced a better response. Unfortunately, due to the small number of cases, the importance of relative ATG dose didn’t show in multifactorial regression analysis, but it is a promising factor when expanding the sample size in the future.

We found that the recovery of ARC and bone marrow megakaryocyte indicated a better response to IST at 12 months, although these patients were evaluated as NR at 6-months. This result is consistent with previous studies. It has been previously reported that ARC at diagnosis is the only independent predictor of OR and FFS at 6 and 12 months in SAA patients, and higher ARC after ATG indicates better bone marrow residual hematopoietic function (12–14). The recovery of peripheral blood erythrocytes and bone marrow megakaryocytes after 6 months also prompted that the bone marrow hematopoiesis recovery, indicating good long-term effects.

TPO-RA is a breakthrough in the history of AA treatment which can not only promote the recovery of megakaryocytes and platelets but also promote HSCs, MPPs and other hematopoietic precursor cells as well as regulate immune system. Studies from the first-line application of EPAG in combination with IST in SAA patients show great efficacy (14–18).The French data in a real-world study showed that the hematologic response rate of EPAG in relapsed/refractory SAA patients was 74%, which was similar to the real-world efficacy of our center (75%) (19). However, TPO-RA did not appear to be the strongest predictor of outcomes at 12 months in multivariate regression analysis in our study, well the ARC at 6 months was the only prognostic factor. In other words, the trend of bone marrow recovery was the independent prognostic factor affecting outcomes at 12 months regardless of treatment. Certainly, the combination of TPO-RA and IST should be used as early as possible as a first-line therapy to achieve early bone marrow and hematologic remission (19). TPO-RA may rescue more SAA patients from secondary ATG or second-line HSCT.

The major limitation of our study is the small sample size, which may be the reason why some variables were meaningful in the univariate analysis but not in the multivariate analysis. The incidence of AA is relatively low, and it is difficult to find the patients who did not get response at 6 months, without receiving secondary ATG or HSCT, but still regular follow-up. We will keep expanding the sample size to update our results.

## Conclusion

For SAA patients who didn’t get response to IST at 6 months, if ATG treatment was performed within 30 days after diagnosis, ATG dosage was sufficient (ATG/lymphocyte ≥2), and ARC was ≥ 30×10^9^/L at 6 months, patients most probably get delayed response and benefit from CsA maintenance. Addition of EPAG could give an even better response. Otherwise, secondary ATG or allo-HSCT treatment were recommended to be given immediately.

## Data availability statement

The original contributions presented in the study are included in the article/**Supplementary Files**, further inquiries can be directed to the corresponding author/s.

## Ethics statement

The studies involving human participants were reviewed and approved by Tianjin Medical University (IRB2022-WZ-169). Written informed consent to participate in this study was provided by the participants’ legal guardian/next of kin.

## Author contributions

CW and TW collected data, analyzed data, and wrote the paper. CL designed the study, analyzed data, and wrote the paper. RF and ZS designed the study and wrote the paper. RF supported experiments. All authors contributed to the article and approved the submitted version. 

## References

[1] Young NS. Aplastic anemia. N Engl J Med (2018) 379(17):1643–56. doi: 10.1056/NEJMra1413485 PMC6467577 30354958

[2] Usuki K. Diagnosis and treatment for aplastic anemia. Rinsho Ketsueki (2021) 62(8):922–30. doi: 10.11406/rinketsu.62.922 34497232

[3] Bacigalupo A. How I treat acquired aplastic anemia. BLOOD (2017) 129(11):1428–36. doi: 10.1182/blood-2016-08-693481 28096088

[4] Red Blood Cell Disease (Anemia) Group Chinese Society of Hematology Chinese Medical Association. Chinese Expert consensus on the diagnosis and treatment of aplastic anemia (2017). Zhong Hua Xue Ye Xue Za Zhi (2017) 38(01):1–5. doi: 10.3760/cma.j.issn.0253-2727.2017.01.001 PMC7348398 28219216

[5] Killick SB Bown N Cavenagh J Dokal I Foukaneli T Hill A. Guidelines for the diagnosis and management of adult aplastic anaemia. Br J Haematol (2016) 172(2):187–207. doi: 10.1111/bjh.13853 26568159

[6] DeZern AE Churpek JE. Approach to the diagnosis of aplastic anemia. Blood Adv (2021) 5(12):2660–71. doi: 10.1182/bloodadvances.2021004345 PMC8270669 34156438

[7] Vallejo C Montesinos P Polo M Cuevas B Morado M Rosell A. Rabbit antithymocyte globulin versus horse antithymocyte globulin for treatment of acquired aplastic anemia: a retrospective analysis. Ann Hematol (2015) 94(6):947–54. doi: 10.1007/s00277-015-2305-3 25672649

[8] Feng X Scheinberg P Biancotto A Rios O Donaldson S Wu C. *In vivo* effects of horse and rabbit antithymocyte globulin in patients with severe aplastic anemia. HAEMATOLOGICA (2014) 99(9):1433–40. doi: 10.3324/haematol.2014.106542 PMC4562531 24907357

[9] Locasciulli A Oneto R Bacigalupo A Socie G Korthof E Bekassy A. Outcome of patients with acquired aplastic anemia given first line bone marrow transplantation or immunosuppressive treatment in the last decade: A report from the European group for blood and marrow transplantation (EBMT). HAEMATOLOGICA (2007) 92(1):11–8. doi: 10.3324/haematol.10075 17229630

[10] Sakamoto T Obara N Kurita N Sakata-Yanagimoto M Nishikii H Yokoyama Y. Effectiveness and safety of rabbit anti-thymocyte globulin in Japanese patients with aplastic anemia. Int J Hematol (2013) 98(3):319–22. doi: 10.1007/s12185-013-1418-5 23963878

[11] Liu L Ding L Hao L Zhang X Li X Zhang L. Efficacy of porcine antihuman lymphocyte immunoglobulin compared to rabbit antithymocyte immunoglobulin as a first-line treatment against acquired severe aplastic anemia. Ann Hematol (2015) 94(5):729–37. doi: 10.1007/s00277-014-2279-6 25604721

[12] Afable MN Shaik M Sugimoto Y Elson P Clemente M Makishima H. Efficacy of rabbit anti-thymocyte globulin in severe aplastic anemia. HAEMATOLOGICA (2011) 96(9):1269–75. doi: 10.3324/haematol.2011.042622 PMC3166096 21606164

[13] Scheinberg P Nunez O Weinstein B Scheinberg P Biancotto A Wu CO. Horse versus rabbit antithymocyte globulin in acquired aplastic anemia. N Engl J Med (2011) 365(5):430–8. doi: 10.1056/NEJMoa1103975 PMC3721503 21812672

[14] Zaimoku Y Patel BA Shalhoub R Groarke EM Feng X Wu CO. Predicting response of severe aplastic anemia to immunosuppression combined with eltrombopag. HAEMATOLOGICA (2022) 107(1):126–33. doi: 10.3324/haematol.2021.278413 PMC8719075 33910334

[15] Townsley DM Scheinberg P Winkler T Desmond R Dumitriu B Rios O. Eltrombopag added to standard immunosuppression for aplastic anemia. N Engl J Med (2017) 376(16):1540–50. doi: 10.1056/NEJMoa1613878 PMC5548296 28423296

[16] Patel BA Groarke EM Lotter J Shalhoub R Gutierrez-Rodrigues F Rios O. Long-term outcomes in patients with severe aplastic anemia treated with immunosuppression and eltrombopag: a phase 2 study. BLOOD (2022) 139(1):34–43. doi: 10.1182/blood.2021012130 34525188 PMC8718619

[17] Li R Zhou J Liu Z Chen X Long Q Yang Y. Predicting response of severe aplastic anemia to rabbit-antithymocyte immunoglobulin based immunosuppressive therapy combined with eltrombopag. Front Immunol (2022) 13:884312. doi: 10.3389/fimmu.2022.884312 35720405 PMC9204341

[18] Isik A Eliacik E Haznedaroglu IC Aksu S Sayinalp N Buyukasik Y. The impact of eltrombopag administration on the clinical course of severe refractory fatal acquired aplastic anemia. Turk J Haematol (2013) 30(3):328–30. doi: 10.4274/Tjh-2013.0005 PMC3878545 24385816

[19] Lengline E Drenou B Peterlin P Tournilhac O Abraham J Berceanu A. Nationwide survey on the use of eltrombopag in patients with severe aplastic anemia: A report on behalf of the French reference center for aplastic anemia. HAEMATOLOGICA (2018) 103(2):212–20. doi: 10.3324/haematol.2017.176339 PMC5792265 29170252

