# Supplementary figures and images for: Partial SAA patients benefit from delayed response of IST
Source: Front Immunol. 2023 Feb 10;14:1067977. doi: 10.3389/fimmu.2023.1067977 (PMC9951814; doi:10.3389/fimmu.2023.1067977)

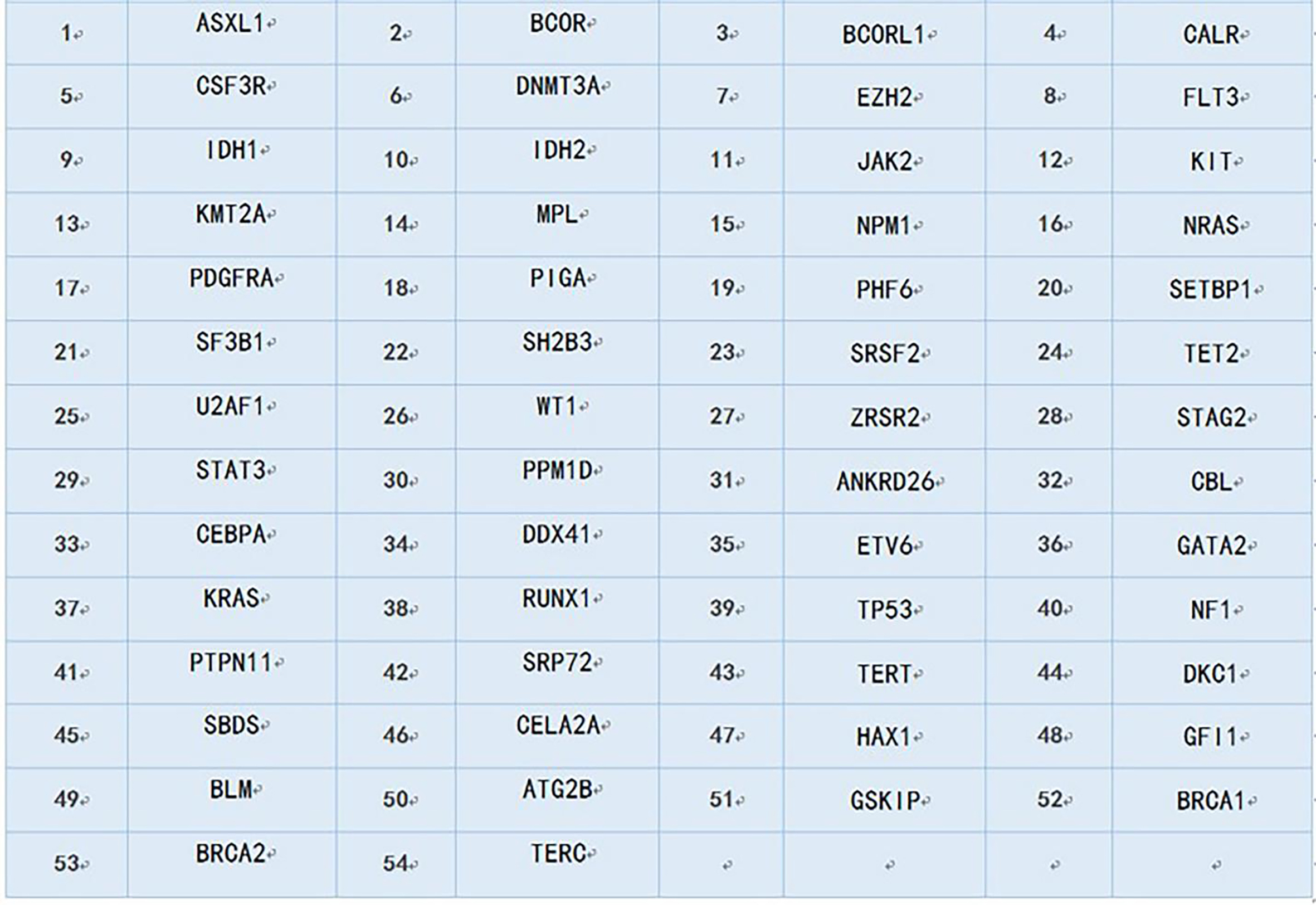

Supplement: Supplementary file 1 [file Image_1.jpeg]
