# Supplementary material for: Partial SAA patients benefit from delayed response of IST
Source: Front Immunol. 2023 Feb 10;14:1067977. doi: 10.3389/fimmu.2023.1067977 (PMC9951814; doi:10.3389/fimmu.2023.1067977)
Supplement: Supplementary file 2 [file Table_1.docx]

**Supplemental**

**Table1. Factors related to the efficacy from 6 to 12 months after rATG with univariate analysis in CsA group.**

| **Covariates** | **RR** | **NR** | **T/Z/χ2** | ***P* value** |
| --- | --- | --- | --- | --- |
| **N (%)** | 11（44%） | 14(56%) |  |  |
| **Age at diagnosis(years)** |  |  | 0.34 | 0.56 |
| **<20** | 6(54.55%) | 6（42.86%） |  |  |
| **≥20** | 5(45.45%) | 8（57.14%） |  |  |
| **Gender** |  |  | 0.37 | 0.55 |
| Male | 9 (81.82%) | 10 (71.43%) |  |  |
| Female | 2 (8.18%) | 4(28.57%) |  |  |
| **ECOG** |  |  | 0.09 | 0.76 |
| **0~1** | 8 (72.73%) | 12(85.71%) |  |  |
| **≥2** | 3 (27.27%) | 2 (14.29%) |  |  |
| **Severity of AA** |  |  | 0.14 | 0.71 |
| **SAA** | 7（63.64%） | 11(78.77%) |  |  |
| **VSAA** | 4（36.36%） | 3(21.23%) |  |  |
| **rATG**[**dosage**](javascript:;)**（mg/kg）** | 4.28±0.78 | 3.59±0.99 | 1.67 | 0.11 |
| **rATG** [**dosage**](javascript:;)**/lymphocytes count(mg🞨kg^-1^/🞨10^9^)** | 2.80(3.32,8.18) | 1.73(1.64,2.49) | -2.46 | **0.014*** |
| **rATG** [**dosage**](javascript:;)**/lymphocytes count(mg🞨kg^-1^/🞨10^9^)** |  |  | 4.26 | **0.04*** |
| **<2** | 3(27.30%) | 8（57.10%） |  |  |
| **≥2** | 8(72.70%) | 6（42.90%） |  |  |
| **Days from diagnosis to IST** | 19.10±5.49 | 50.08±45.32 | -2.58 | **0.022*** |
| **Days from diagnosis to IST** |  |  | 9.29 | **0.002*** |
| **<30days** | 10（90.90%） | 3（21.40%） |  |  |
| **≥30days** | 1（9.10%） | 11（78.60%） |  |  |
| **The indicators before IST as follows** | |  |  |  |
| **WBC,🞨10^9^/L** | 1.28±1.25 | 2.18±1.98 | -0.42 | 0.68 |
| **ANC,🞨10^9^/L** | 0.14±0.34 | 0.15±0.16 | -0.60 | 0.56 |
| **ALC,🞨10^9^/L** | 1.19±0.88 | 1.79±0.66 | -2.14 | 0.06 |
| **HB,g/L** | 73.33±20.68 | 75.43±23.84 | 0.96 | 0.35 |
| **RBC,🞨10^12^/L** | 2.36±0.64 | 2.39±0.80 | 1.06 | 0.30 |
| **PLT,🞨10^9^/L** | 20.50±21.35 | 16.00±6.43 | 0.19 | 0.85 |
| **ARC,🞨10^9^/L** | 6.45±4.05 | 8.39±8.93 | 0.06 | 0.96 |
| **CD4^+^T cells(%)** | 33.78±14.61 | 35.11±16.52 | -0.74 | 0.47 |
| **CD4^+^T cells, 🞨10^9^/L** | 0.44±0.35 | 0.63±0.30 | -1.59 | 0.13 |
| **CD8^+^T cells (%)** | 38.65±13.78 | 44.96±10.48 | -1.50 | 0.15 |
| **CD8^+^T cells, 🞨10^9^/L** | 0.46±0.37 | 0.80±0.18 | -2.93 | **0.008*** |
| **CD4/CD8** | 1.06±0.65 | 1.01±0.46 | -0.09 | 0.93 |
| **CD19^+^B cells（%）** | 16.64±14.26 | 17.24±13.21 | -0.87 | 0.40 |
| **CD5^+^CD19^+^B cells (%)** | 23.15±16.37 | 34.07±7.58 | -0.64 | 0.53 |
| **mDC cells（%）** | 0.05(0.01,0.26) | 0.09(0.03,0.63) | -1.92 | 0.055 |
| **pDC cells（%）** | 0.06(0.03,0.48) | 0.03(0.02,0.27) | -0.32 | 0.75 |
| **mDC /pDC** | 0.67(0.5,1.44) | 1.67(1.29,2.70) | -2.82 | **0.005*** |
| **Initial IL-2(pg/ml)** | 5.55(3.07,5.81) | 5.75(3.90,6.66) | -0.76 | 0.45 |
| **Initial IL-4(pg/ml)** | 0.67(0.34,23.55) | 3.17(0.42,39.53) | -0.49 | 0.62 |
| **Initial IL-6(pg/ml)** | 4.41(3.03,7.0) | 4.72(4.09,7.96) | -0.37 | 0.71 |
| **Initial IL-10(pg/ml)** | 3.12(1.11,5.59) | 5.25(3.42,5.95) | -0.60 | 0.55 |
| **Initial TNF-a(pg/ml)** | 3.28(1.89,3.76) | 4.71(2.54,6.41) | -1.06 | 0.29 |
| **Initial IFN-r(pg/ml)** | 3.07(1.59,4.86) | 1.91(0.87,4.26) | -0.86 | 0.39 |
| **Myeloid cell in bone marrow (%)** | 22.50(6.0,33.5) | 11.0(6.75,26.63) | -0.47 | 0.64 |
| **Erythrocyte in bone marrow（%）** | 8.0(1.50,19.0) | 5.25(0,16.13) | -0.39 | 0.70 |
| **megakaryocytes** | 0 | 0 |  | 0.97 |
| **The indicators at 6months after IST as follows** | | | | |
| **WBC, 🞨10^9^/L** | 5.65±1.66 | 6.91±1.66 | 0.37 | 0.72 |
| **ANC,🞨10^9^/L** | 1.11±1.60 | 3.89±3.24 | -0.91 | 0.37 |
| **ALC,🞨10^9^/L** | 0.95±0.87 | 0.98±0.42 | -1.52 | 0.15 |
| **HB,g/L** | 669.73±7.59 | 57.33±9.29 | 2.78 | **0.011*** |
| **RBC,🞨10^12^/L** | 2.62±0.43 | 1.84±0.37 | 2.001 | 0.057 |
| **PLT,🞨10^9^/L** | 19.80±5.12 | 24.17±14.15 | 1.30 | 0.21 |
| **ARC,🞨10^9^/L** | 63.53±26.19 | 27.30±19.68 | 2.33 | **0.029*** |
| **ARC,🞨10^9^/L** |  |  | 7.35 | **0.007*** |
| **<30** | 1（9.1%） | 10（71.4%） |  |  |
| **≥30** | 10（90.9%） | 4（28.6%） |  |  |
| **CD4^+^T cells (%)** | 21.46±12.83 | 15.93±4.44 | 0.69 | 0.50 |
| **CD4^+^T cells,🞨10^9^/L** | 0.21±0.14 | 0.17±0.10 | 0.26 | 0.80 |
| **CD8+T cells (%)** | 50.0±16.60 | 53.76±13.18 | -0.91 | 0.38 |
| **CD8+T cells,🞨10^9^/L** | 0.44±0.14 | 0.53±0.23 | -1.15 | 0.27 |
| **CD4/CD8** | 0.48±0.31 | 1.30±2.43 | -0.70 | 0.49 |
| **CD19^+^B cells（%）** | 8.87±6.35 | 4.14±3.25 | 1.62 | 0.14 |
| **CD5^+^CD19^+^B cells（%）** | 27.60±11.77 | 16.73±10.01 | 1.66 | 0.13 |
| **mDC（%）** | 0.08±0.01 | 0.16±0.18 | 1.03 | 0.35 |
| **pDC（%）** | 0.11±0.02 | 0.11±0.05 | 1.05 | 0.34 |
| **mDC /pDC** | 0.72±0.11 | 3.10±5.10 | -0.28 | 0.78 |
| **IL-2（pg/ml）** | 2.74±0.59 | 3.39±1.40 | -2.16 | 0.08 |
| **IL-4（pg/ml）** | 1.09±0.99 | 1.28±1.41 | -0.51 | 0.62 |
| **IL-6（pg/ml）** | 2.73±3.66 | 4.24±1.51 | -1.93 | 0.86 |
| **IL-10（pg/ml）** | 2.92±1.75 | 1.65±0.73 | -0.73 | 0.48 |
| **TNF-a（pg/ml）** | 3.30±4.09 | 1.99±1.48 | 6.63 | 0.54 |
| **IFN-r（pg/ml）** | 6.23±6.34 | 3.12±3.53 | 0.74 | 0.48 |
| **Myeloid cell in bone marrow (%)** | 72.75±9.55 | 73.63±8.38 | 0.08 | 0.94 |
| **Erythrocyte in bone marrow（%）** | 15.75±8.83 | 12.13±9.0 | 1.48 | 0.15 |
| [**megakaryocyte**](javascript:;) | 10.5(4.0,23.75) | 3.0(0,12.0) | -2.82 | **0.005*** |

IST, immunosuppressive therapy; ALC, absolute lymphocyte count; ANC, absolute neutrophil count; mDC, myeloid dendritic cell; pDC, plasmacytoid dendritic cell; ARC, absolute reticulocyte count; HB, hemoglobin; red blood cell; RBC, red blood cell; PLT, platelet;
