# Supplementary material for: Partial SAA patients benefit from delayed response of IST
Source: Front Immunol. 2023 Feb 10;14:1067977. doi: 10.3389/fimmu.2023.1067977 (PMC9951814; doi:10.3389/fimmu.2023.1067977)
Supplement: Supplementary file 3 [file Table_2.docx]

**Supplemental**

**Table2. Factors related to the efficacy from 6 to 12 months after rATG with univariate analysis in CsA and EPAG group.**

| **Covariates** | **RR** | **NR** | **T/Z/χ2** | ***P* value** |
| --- | --- | --- | --- | --- |
| **N (%)** | 15（75.0%） | 5(25.0%) |  |  |
| **Age at diagnosis(years)** |  |  | 1.27 | 0.26 |
| **<20** | 3(20.0%) | 3（60.0%） |  |  |
| **≥20** | 12(80.0%) | 2（40.0%） |  |  |
| **Gender** |  |  | 0.31 | 0.61 |
| Male | 11 (73.3%) | 3 (60.0%) |  |  |
| Female | 4 (26.7%) | 2(40.0%) |  |  |
| **ECOG** |  |  | 1.83 | 0.11 |
| **0~1** | 8 (53.3%) | 5(100%) |  |  |
| **≥2** | 7 (46.7%) | 0 |  |  |
| **Severity of AA** |  |  | 0.27 | 1.0 |
| **SAA** | 7(46.7%) | 3 (60.0%) |  |  |
| **VSAA** | 8 (53.3%) | 2(40.0%) |  |  |
| **rATG**[**dosage**](javascript:;)**（mg/kg）** | 3.21±0.68 | 3.68±0.71 | -1.21 | 0.24 |
| **rATG** [**dosage**](javascript:;)**/lymphocytes count(mg🞨kg^-1^/🞨10^9^)** | 2.30±0.98 | 2.09±0.34 | 0.41 | 0.69 |
| **rATG** [**dosage**](javascript:;)**/lymphocytes count(mg🞨kg^-1^/🞨10^9^)** |  |  | 0.004 | 0.95 |
| **<2** | 6(42.9%) | 1（25.0%） |  |  |
| **≥2** | 8(57.1%) | 3（75.0%） |  |  |
| **Days from diagnosis to IST** | 21(15,30) | 21(13,29) | -0.61 | 0.54 |
| **Days from diagnosis to IST** |  |  | 0.14 | 1.0 |
| **<30** | 10（71.4%） | 4（80.0%） |  |  |
| **≥30** | 4（28.6%） | 1（20.0%） |  |  |
| **The indicators before IST as follows** | |  |  |  |
| **WBC,🞨10^9^/L** | 1.55±0.47 | 1.13±1.09 | 0.85 | 0.44 |
| **ANC,🞨10^9^/L** | 0.13(0.07,0.48) | 0.30(0.02,0.62) | -0.22 | 0.83 |
| **ALC,🞨10^9^/L** | 1.61(1.52,1.65) | 1.76(1.71,1.87) | -3.27 | 0.001* |
| **HB,g/L** | 71.20±6.27 | 75.40±3.65 | -1.41 | 0.18 |
| **RBC,🞨10^12^/L** | 2.21(1.97,2.45) | 2.21(2.17,2.54) | -0.79 | 0.43 |
| **PLT,🞨10^9^/L** | 21.53±13.07 | 17.40±6.66 | 0.67 | 0.51 |
| **ARC,🞨10^9^/L** | 12.85±8.75 | 6.44±5.63 | 1.52 | 0.15 |
| **CD4^+^T cells (%)** | 36.21±15.24 | 43.07±11.42 | -0.83 | 0.42 |
| **CD4^+^T cells, 🞨10^9^/L** | 0.52±0.21 | 0.78±0.22 | -2.08 | 0.055 |
| **CD8^+^T cells (%)** | 42.6（29.5，65.40） | 30.0（25.5，30.49） | -1.48 | 0.14 |
| **CD8^+^T cells, 🞨10^9^/L** | 0.68±0.33 | 0.52±0.07 | 0.96 | 0.35 |
| **CD4/CD8** | 1.15±0.79 | 1.39±0.47 | -0.64 | 0.53 |
| **CD19^+^B cells（%）** | 19.34±16.10 | 13.95±10.79 | 0.58 | 0.58 |
| **CD5^+^CD19^+^B cells (%)** | 18.10±10.66 | 22.89±8.80 | -0.74 | 0.48 |
| **Myeloid cell in bone marrow (%)** | 9.0(5.0,12.0) | 3.0(1.25,5.0) | -2.28 | 0.23 |
| **Erythrocyte in bone marrow（%）** | 2.0(1.0,12.0) | 1.0(0,1.75) | -1.99 | 0.05 |
| **megakaryocytes** | 0 | 0 | -0.84 | 0.40 |
| **The date at 6months after IST as follows** | | | | |
| **WBC, 🞨10^9^/L** | 7.82±5.40 | 8.72±6.81 | -0.30 | 0.77 |
| **ANC,🞨10^9^/L** | 3.69(0.14,10.28) | 4.21(0.03,13.20) | -0.57 | 0.57 |
| **ALC,🞨10^9^/L** | 1.05±0.56 | 1.25±0.78 | -0.63 | 0.54 |
| **HB,g/L** | 70.40±6.22 | 75.40±3.65 | -1.68 | 0.11 |
| **RBC,🞨10^12^/L** | 2.25±0.33 | 2.32±0.20 | -0.44 | 0.66 |
| **PLT,🞨10^9^/L** | 17.73±7.29 | 21.40±7.13 | -0.98 | 0.34 |
| **ARC,🞨10^9^/L** | 55.90±30.77 | 31.85±25.89 | 1.57 | 0.14 |
| **ARC,🞨10^9^/L** |  |  | 0.32 | 0.61 |
| **<30** | 4（26.7%） | 2（40.0%） |  |  |
| **≥30** | 11（73.3%） | 3（60.0%） |  |  |
| **CD4^+^T cells (%)** | 42.71±18.66 | 25.11±14.01 | 1.91 | 0.07 |
| **CD4^+^T cells,🞨10^9^/L** | 0.37(0.18,0.66) | 0.54(0.06,0.61) | -0.83 | 0.41 |
| **CD8^+^T cells (%)** | 25.35(21.0,47.64) | 44.0(30.12,64.90) | -1.02 | 0.31 |
| **CD8^+^T cells,🞨10^9^/L** | 0.37±0.41 | 0.51±0.35 | -0.67 | 0.51 |
| **CD4/CD8** | 1.68±1.18 | 0.37±0.35 | 2.40 | 0.03 |

IST, immunosuppressive therapy; ALC, absolute lymphocyte count; ANC, absolute neutrophil count; mDC, myeloid dendritic cell; pDC, plasmacytoid dendritic cell; ARC, absolute reticulocyte count; HB, hemoglobin; red blood cell; RBC, red blood cell; PLT, platelet;
