# Supplementary material for: Partial SAA patients benefit from delayed response of IST
Source: Front Immunol. 2023 Feb 10;14:1067977. doi: 10.3389/fimmu.2023.1067977 (PMC9951814; doi:10.3389/fimmu.2023.1067977)
Supplement: Supplementary file 4 [file Table_3.docx]

**Supplemental**

**Table3. Factors related to the efficacy from 6 to 12 months after rATG with univariate analysis in all patients.**

| **Covariates** | **RR** | **NR** | **T/Z/χ2** | ***P* value** |
| --- | --- | --- | --- | --- |
| **Age at diagnosis** |  |  | 0.74 | 0.39 |
| <20 | 9(34.62%) | 9（47.37%） |  |  |
| ≥20 | 17(65.38%) | 10(52.63%） |  |  |
| **Gender** |  |  | 0.41 | 0.52 |
| Male | 20(76.92%) | 13 (68.42%) |  |  |
| Female | 6(23.08%) | 6(31.58%) |  |  |
| **ECOG** |  |  | 3.07 | 0.08 |
| 0~1 | 16 (61.50%) | 17(89.50%) |  |  |
| ≥2 | 10 (38.50%) | 2 (10.50%) |  |  |
| **Severity of AA** |  |  | 1.84 | 0.18 |
| SAA | 14（53.80%） | 14(73.70%) |  |  |
| VSAA | 12(46.20%） | 5(26.30%) |  |  |
| **TPO-RA** |  |  | 4.38 | **0.04*** |
| **Yes** | 15(57.70%) | 5(26.30%) |  |  |
| **No** | 11(42.30%) | 14(73.70%) |  |  |
| **rATG** [**dosage**](javascript:;)**（mg/kg）** | 3.67±0.90 | 3.61±0.91 | 0.34 | 0.73 |
| **rATG** [**dosage**](javascript:;)**/lymphocytes count(mg🞨kg^-1^/🞨10^9^)** | 2.28(1.94,3.08) | 1.75(1.64,2.41) | -1.92 | 0.055 |
| **rATG** [**dosage**](javascript:;)**/lymphocytes count(mg🞨kg^-1^/🞨10^9^)** |  |  | 3.09 | 0.079 |
| **<2.0** | 7（26.90%） | 10（52.60%） |  |  |
| **≥2.0** | 19（73.10%） | 9（47.40%） |  |  |
| **Days from diagnosis to IST** | 20.21±6.92 | 41.68±39.50 | -2.34 | **0.03*** |
| **Days from diagnosis to IST** |  |  | 9.01 | **0.03*** |
| **<30** | 21(80.8%) | 7(36.8%) |  |  |
| **≥30** | 5(19.2%) | 12(63.2%) |  |  |
| **The indicators before IST as follows** | |  |  |  |
| **WBC,🞨10^9^/L** | 1.41±0.89 | 1.66±1.48 | 0.36 | 0.73 |
| **ANC,🞨10^9^/L** | 0.13(0,0.46) | 0.30(0.03,0.43) | -0.85 | 0.40 |
| **ALC,🞨10^9^/L** | 0.44±0.69 | 0.61±0.85 | -0.44 | 0.67 |
| **HB,g/L** | 74.42±18.57 | 74.65±17.20 | 0.10 | 0.92 |
| **RBC,🞨10^12^/L** | 2.28(1.91,2.87) | 2.19(1.99,2.59) | -0.09 | 0.93 |
| **PLT,🞨10^9^/L** | 19.16±14.98 | 14.00±6.59 | 0.15 | 0.88 |
| **ARC,🞨10^9^/L** | 8.72±5.35 | 8.40±8.46 | 1.34 | 0.19 |
| **CD4^+^T cells (%)** | 36.07±17.35 | 40.68±15.01 | -0.03 | 0.98 |
| **CD4^+^Tcells, 🞨10^9^/L** | 0.48±0.30 | 0.72±0.28 | -1.41 | 0.18 |
| **CD8^+^T cells (%)** | 38.82±15.15 | 40.52±12.09 | -0.75 | 0.47 |
| **CD8^+^T cells, 🞨10^9^/L** | 0.61±0.35 | 0.73±0.23 | -2.35 | **0.03*** |
| **CD4/CD8** | 1.12±0.78 | 1.19±0.54 | -0.03 | 0.98 |
| **CD19^+^B cells（%）** | 16.25±13.41 | 17.72±12.79 | 0.014 | 0.98 |
| **CD5^+^CD19^+^B cells (%)** | 23.55±13.55 | 29.20±15.32 | -1.38 | 0.19 |
| **mDC cells（%）** | 0.17(0.04,0.82) | 0.08(0.04,0.52) | -0.90 | 0.37 |
| **pDC cells（%）** | 0.07(0.04,0.76) | 0.04(0.03,0.26) | -1.62 | 0.11 |
| **mDC /pDC** | (1.13±0.84) | (1.91±1.36) | -1.46 | 0.16 |
| **IL-2（pg/ml）** | 4.68±2.88 | 5.57±3.63 | -0.65 | 0.55 |
| **IL-4（pg/ml）** | 2.30(0.37,7.45) | 5.25(1.09,7.06) | -0.59 | 0.56 |
| **IL-6（pg/ml）** | 5.13±2.46 | 5.21±2.20 | 0.33 | 0.95 |
| **IL-10（pg/ml）** | 4.07(1.57,5.38) | 5.25(2.97,12.41) | -1.11 | 0.27 |
| **TNF-a（pg/ml）** | 3.69±1.74 | 3.88±1.64 | -0.57 | 0.81 |
| **IFN-r（pg/ml）** | 2.29(1.11,3.12) | 2.64(1.75,8.24) | 0.54 | 0.59 |
| **Myeloid cell in bone marrow (%)** | 10.25(5.75,26.50) | 7.0(3.0,17.5) | -0.82 | 0.41 |
| **Erythrocyte in bone marrow (%)** | 3.0(1.0,14.13) | 1.0(0,15.5) | -1.04 | 0.30 |
| [**megakaryocyte**](javascript:;) | 0 | 0 | -0.08 | 0.84 |
| **The indicators at 6months after IST as follows** | | | | |
| **WBC, 🞨10^9^/L** | 7.43±4.62 | 7.03±4.19 | 0.34 | 0.77 |
| **ANC,🞨10^9^/L** | 2.06(0,7.27) | 4.16(0.18,7.69) | -0.85 | 0.46 |
| **ALC,🞨10^9^/L** | 0.37(0,0.98) | 0.71(0,1.36) | 0.40 | 0.18 |
| **HB, g/L** | 70.12±6.70 | 63.16±12.23 | 2.25 | 0.03 |
| **RBC,🞨10^12^/L** | 2.21±0.33 | 1.20±0.38 | 2.06 | 0.046 |
| **PLT,🞨10^9^/L** | 25.73±20.33 | 19.68±12.51 | 1.16 | 0.26 |
| **ARC,🞨10^9^/L** | 56.77±30.37 | 28.78±30.56 | 3.04 | **0.004*** |
| **ARC,🞨10^9^/L** |  |  | 8.95 | **0.003*** |
| **<30** | 4（15.4%） | 12（63.20%） |  |  |
| **≥30** | 22（84.60%） | 7（36.80%） |  |  |
| **CD4^+^T cells (%)** | 23.58±9.10 | 22.10±10.62 | 0.15 | 0.69 |
| **CD4^+^T cells,🞨10^9^/L** | 0.35±0.24 | 0.29±0.20 | -0.33 | 0.75 |
| **CD8^+^T cells (%)** | 50.40±13.45 | 53.26±16.27 | -0.90 | 0.61 |
| **CD8^+^T cells,🞨10^9^/L** | 0.43±0.34 | 0.59±0.29 | 1.40 | 0.19 |
| **CD4/CD8** | 0.38(0.32,0.76) | 0.38(0.20,0.62) | -0.50 | 0.62 |
| **CD19^+^B cells（%）** | 5.73±5.89 | 4.95±4.17 | 0.28 | 0.78 |
| **CD5^+^CD19^+^B cells（%）** | 26.42±10.36 | 18.52±11.10 | 1.91 | 0.14 |
| **mDC（%）** | 0.21(0.32,0.76) | 0.12(0.08,0.15) | -1.51 | 0.14 |
| **pDC（%）** | 0.18(0.11,0.52) | 0.11(0.05,0.17) | -1.77 | 0.08 |
| **mDC /pDC** | 1.57(0.91,2.63) | 0.80(0.44,2.50) | -1.16 | 0.26 |
| **IL-2（pg/ml）** | 2.12±1.06 | 3.13±1.54 | -1.85 | 0.07 |
| **IL-4（pg/ml）** | 2.05±1.93 | 1.56±1.45 | 0.16 | 0.52 |
| **IL-6（pg/ml）** | 2.93±1.57 | 4.04±1.81 | -2.35 | 0.14 |
| **IL-10（pg/ml）** | 2.64±1.92 | 3.20±2.47 | -0.72 | 0.56 |
| **TNF-a（pg/ml）** | 2.42±1.76 | 1.71±1.27 | 0.70 | 0.30 |
| **IFN-r（pg/ml）** | 2.74(1.02,6.96) | 1.93(0.77,6.13) | -0.41 | 0.68 |
| **Myeloid cell in bone marrow (%)** | 68.08±10.55 | 62.07±19.91 | 0.03 | 0.47 |
| **Erythrocyte in bone marrow (%)** | 17.44±11.88 | 14.57±10.61 | 0.15 | 0.28 |
| [**megakaryocyte**](javascript:;) | 8.5(0,24.0) | 2.5(0,5.0) | -1.85 | 0.07 |

IST, immunosuppressive therapy; ALC, absolute lymphocyte count; ANC, absolute neutrophil count; mDC, myeloid dendritic cell; pDC, plasmacytoid dendritic cell; ARC, absolute reticulocyte count; HB, hemoglobin; red blood cell; RBC, red blood cell; PLT, platelet;
